# Forced Expiratory Flow at 25–75% as a Marker for Airway Hyper Responsiveness in Adult Patients with Asthma-like Symptoms

**Published:** 2018-02

**Authors:** Hanieh Raji, Maryam Haddadzadeh Shoushtari, Esmaeil Idani, Heshmatollah Tavakol, Sakineh Afrakhteh, Maryam Dastoorpoor, Seyed Hamid Borsi

**Affiliations:** 1 Air pollution and Respiratory Diseases Research Center, Ahvaz Jundishapur University of Medical Sciences, Ahvaz, Iran; 2 Department of Epidemiology and Biostatistics, School of Public Health, Ahvaz Jundishapur University of Medical Sciences, Ahvaz, Iran

**Keywords:** Airway Hyper-Responsiveness, Methacholine Challenge Test, Asthma, FEF_25-75_

## Abstract

**Background:**

The aim of the present study was threefold: to assess the association between baseline FEF_25-75_ and Airway Hyper-responsiveness (AHR), to specify whether a decrease in FEF_25-75_ may reflect severe hyper-responsiveness, and finally to confirm a FEF _25-75_ cut-off value.

**Materials and Methods:**

In a cross sectional study in Imam Khomeini Hospital, Ahvaz, patients suffering from respiratory symptoms due the 2013 autumn rainfall with normal FEV1 and FEV1/FVC were evaluated by methacholine challenge test. Those with PD20<1000, 1000<PD20<2000 or >2000 μg were classified as severe, moderate and mild AHR, respectively. Data were analyzed using Chi-square, Independent t-test, One-way ANOVA and Receiver Operating Characteristic (ROC) curve.

**Results:**

Among the 234 patients, mean baseline FEF_25-75_ was 84.2±22.7% for 54 patients having a negative bronchial provocation test result and 70.9±19.2% for 179 patients with a positive bronchial provocation test result (P < 0.0001). No change was observed in the median PD20 among patients with a higher baseline FEF_25-75_. ROC analysis showed that FEF_25-75_ could potentially be a predictor of AHR, but it could not confirm the cut-off value of FEF_25-75_ for these patients.

**Conclusion:**

When asthma begins, AHR could be predicted by impaired FEF_25-75_ with normal FEV1 and FEV1/FVC. However, we could not determine a cut-off value, and no association was found between a greater impairment of FEF_25-75_ and a more severe AHR.

## INTRODUCTION

Bronchial hyper-responsiveness is defined as an abnormal bronchial response to stimulants and it has been considered as a typical characteristic of chronic asthma (1). One of the most important parameters for the diagnosis and post-diagnosis follow-up of asthma is the Forced Expiratory Volume in 1 second (FEV_1_). However, recent studies have demonstrated that asthmatic patients with a normal FEV1 may have ventilatory defects (2) and suggest another parameter which is the expiratory flow between 25% and 75% of vital capacity (FEF_25-75_) which is more reflective of small airways and a sensitive indicator of symptomatic asthma, compared to FEV_1_, in detecting airways limitation (2–4). What distinguishes FEF_25-75_ from FEV1 is the fact that the recorded values in the latter are concerned with the whole bronchial tree, while the former provides values specifically related to the bronchial zone between division 7 and division 19, the internal diameter of which is between 0.5 and 2 mm. In addition, whereas the values of FEV1 are more reliable in showing the degree of bronchial obstruction, FEF_25-75_ is more variable and sometimes used when FEV1 is within normal limits (5,6). Since small airways are more susceptible to inflammatory and remodeling processes, it is important to determine whether FEF_25-75_ is a preferred tool in assessing AHR when the methacholine challenge test is performed (7,8). The combination of a low FEF_25-75_ and a normal FEV_1_ as a hallmark of asthma is not yet well established (4). No guidelines have been offered as to find normal FEF_25-75_ values. In this regard, a FEF_25-75_ cut-off value has recently been proposed for a group of asthmatic children: FEF_25-75_ less than 65% of predicted is considered impaired (3). Impaired FEF_25-75_ may be suggestive of severe bronchial hyper-reactivity in patients with recent onset of allergic rhinitis. A positive response to bronchodilator and an underlying bronchial inflammation can be assessed by Fractional exhaled Nitric oxide (FeNO) measurement (9). The Methacholine Challenge Test (MCT) has been used universally to assess bronchial hyper-responsiveness in patients with asthma. Although MCT is as a standard method to confirm the presence of airway hyper-responsiveness, it has its own limitations (in available and cost of procedure) that restrict its use as a tool for definitive diagnosis of asthma (10). Consecutive methacholine doses are administered until FEV_1_ is seen to decrease by 20 percent (PD20) (11).

The current study was designed to assess the presence of Airway Hyper-responsiveness (AHR) in a large group of adults suffering from an acute rainfall dyspnoea, to examine the relationship between FEF_25-75_ and methacholine airway responsiveness, to confirm a cut-off value for FEF_25-75_ in these patients and determine a relationship between baseline FEF_25-75_ and AHR. More specifically, the relationship between a greater impairment of FEF_25-75_ and a more severe AHR was aimed to be investigated.

## MATERIALS AND METHODS

### Patients and Study Design

The present study was a cross-sectional study carried out at the Department of Pulmonology, Imam Khomeini Hospital, Ahvaz, Iran. It was approved by Ahvaz University of Medical Sciences with DU-9302 number. In this study, 236 adults with asthma-like symptoms due to the 2013 autumn rainfall and with normal FEV_1_ and FEV_1_ / FVC were evaluated by performing the methacholine challenge test. In fact, since all patients had asthma-like symptoms (e.g., inexplicable acute attacks of cough, wheezing, dyspnea, etc.) due to rainfall with a normal spirometry, they were subject to a methacholine challenge test. The use of spirometer and methacholine challenge test data was approved by the Ethics Committee of Ahvaz Jundishapur University of Medical Sciences.

The patients with normal respiratory function with FEV1/FVC > 70% were included. Exclusion criteria contained failure to perform spirometry with an acceptable quality, history of heart attack or stroke within the last 3 months, uncontrolled hypertension (systolic BP > 200 mmHg, or diastolic BP > 100 mmHg), current use of corticosteroid, beta agonist, anticholinergic, theophylline, antileukotriene, chromones, beta-blocker and cholinesterase inhibitor medication (for myasthenia gravis), pregnancy, and unwillingness to participate. A standard questionnaire was used to record demographic details such as age, sex, spirometry and methacholine test results. All participants signed a written informed consent.

### Spirometry

All participants underwent basal spirometry. The patients’ pulmonary function report involved their age, gender, weight, height, and smoking status. Abiding by ATS/ERS guidelines (8) regarding the standards of lung function testing, spirometric assessment was conducted using a spirometer (Ganshorn medizin electronic) and the best test is defined as the best FVC, FEV1 and FEF_25-75_ of all the reproducible tests; these data also were used to calculate FEV1/FVC ratio.

The patients’ pulmonary function variables were expressed in relation to the amount predicted for healthy subjects with similar age, weight, and height. In order to examine the hypothetical relationship between FEF_25-75_ and AHR, the subjects were subdivided into 3 groups according to the baseline FEF_25-75_%: FEF_25-75_≤50% or FEF_25-75_>50 and ≤65% or FEF_25-75_>65%.

### Methacholine Bronchial Provocation Protocol

The tests were conducted at the Pulmonary Department of Imam Khomeini Hospital. Methacholine solutions were prepared using dry methacholine powder based on aseptic technique by trained personnel. A methacholine concentration of 0.06 mg/ml to 16 mg/ml was diluted in normal saline (0.9% sodium chloride). The patients received the solutions via an Aerosol-Dosimeter ProvoJet (Ganshorn Medizin Electronic).

After baseline spirometry, methacholine was inhaled according to ATS guidelines where a 2-min tidal breathing method was used with a synchronized nebulizer (12). Nebulized methacholine was inhaled for 2 minutes, and there were 5-minute intervals between doses. Seven inhalations of increasing concentrations of methacholine were administered, namely 0.06, 0.125, 0.5, 1, 4, 8 and 16 mg/ml, until the highest concentration (16 mg/ml) or the end-point (a 20% decrease in FEV_1_) was reached. Spirometry was performed 3 minutes after each increasing dose of methacholine. Patients in whom a 20% fall in FEV1 with a methacholine dose of 16 mg/ml was not observed, were considered normal.

Subjects were divided into three groups based on the extent of methacholine required to attain the aforesaid parameters: Group 1 (Severe):≤1000μg; Group 2 (Moderate): 1001–2000μg; and Group 3 (Mild):≥2001μg (11). They were also classified according to FEF_25-75_ into three groups: FEF<50, 50<FEF<65 and FEF>65.

### Statistical analysis

Kolmogorov-Smirnov test was employed to determine whether sample data were normally distributed (p.value>0.05). Data analysis was performed using descriptive statistics such as frequency, frequency percentage, mean and standard deviation. Statistical inferences were made based on different tests including independent t-test, Chi-square, and one-way ANOVA.

A Receiver-Operating Characteristic (ROC) curve analysis was conducted to evaluate the ability of FEF_25-75_% to predict airway AHR. A p-value of less than 0.05 was considered statistically significant. Statistical analysis specificity was performed using SPSS version 22.0.

## RESULTS

Of the 236 patients with asthma-like symptoms, 113 (47.9 %) were male and 123 (52.1 %) were female. Average age of the subjects was 28.4 ± 12.3 years (range: 23–64). About 13.7% of the patients were smokers. Pervious history of asthma was positive in 26.1%. Methacholine challenge test results were positive for 182 (77.1%) subjects and negative for 54 (22.9%).

Mean FEF_25-75_ value was 70.9 ± 19.2 and 84.2 ± 22.7, for patients with positive and negative Methacholine challenge test result, respectively, and the difference was statistically significant (t = 4.003, p-value<0.001). In other words, mean FEF_25-75_ value is lower for patients with hyper-responsiveness airways.

**Figure 1.**
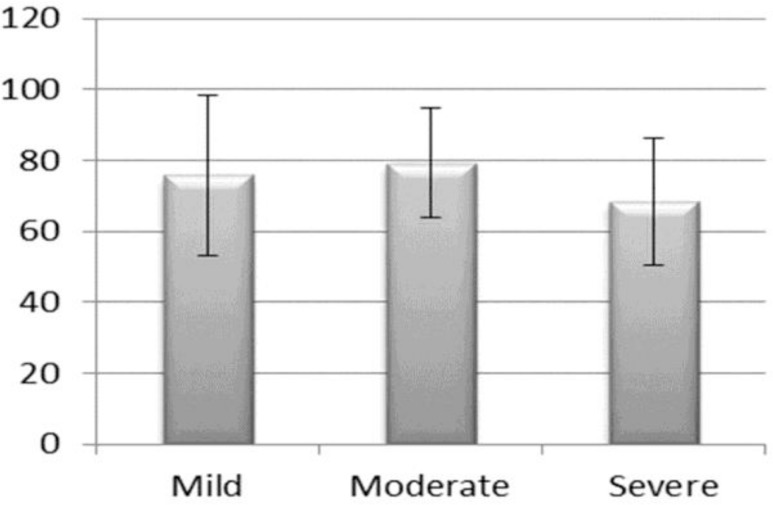
Mean distribution of FEF_25-75_ for hyper-responsiveness level.

Later on all positive methacholine test subjects were subdivided in groups on the basis of bronchial hyper-responsiveness (≤ 1000 μg, between 1001–2000 μg; and ≥ 2001 μg), one-way ANOVA results indicated that mean FEF_25-75_ value was significantly lower for severe bronchial hyper-responsiveness group (F=3.78, df=3, P-value = 0.025; Figure-1).

Frequency of people getting either a negative or positive methacholine test was significantly different for different groups based on their FEF_25-75_ values (p-value=0.01) (Table 1). When we subdivided all patients in groups according to the baseline FEF_25-75_ values (<50%, between 50 and 65% and >65%), the values of positive methacholine test decreased significantly when going from FEF_25-75_<50% to values >65% (Table 1). Also these percentages for negative tests were converse.

**Table 1.** Frequency distribution of methacholine test results for different FEF_25-75_ values (n=234)

	**FEF 25–75%**			
**Methacholine Test**	≤50	51–65	>65	P-value
**Negative**	3(11.1%)	8(13.3%)	44(29.9%)	0.01^¥^
**Positive**	24(88.9%)	52(86.7%)	103(70.1%)	
**Total**	27(100%)	60(100%)	147(100%)	

¥ Chi-squared test

In addition, frequency distribution of hyper-responsiveness had an interesting pattern. The group with severe hyper-responsiveness had the highest frequency, as illustrated in table 2. However, among patients with mild, moderate and severe AHR, there was no significant difference when going from baseline values of FEF_25-75_<50% to values >65% with the increase of baseline FEF_25-75_%. In addition, no change in the median PD20 was observed among patients whose baseline FEF_25-75_% was higher.

**Table 2.** Frequency distribution of hyper-responsiveness level for different FEF_25-75_ values (n=179)

**Hyper-responsiveness**	**<50%**	**51–65%**	**>65%**	**P-value**
**Mild**	6(25%)	8(15.4%)	29(28.2%)	
**Moderate**	1 (4.2%)	2(3.8%)	11(10.7%)	0.14^¥^
**Severe**	17(70.8%)	42 (80.8%)	63(61.2%)	
**Total**	24(100%)	52(100%)	103(100)	

¥ Chi-squared test

**Figure 2.**
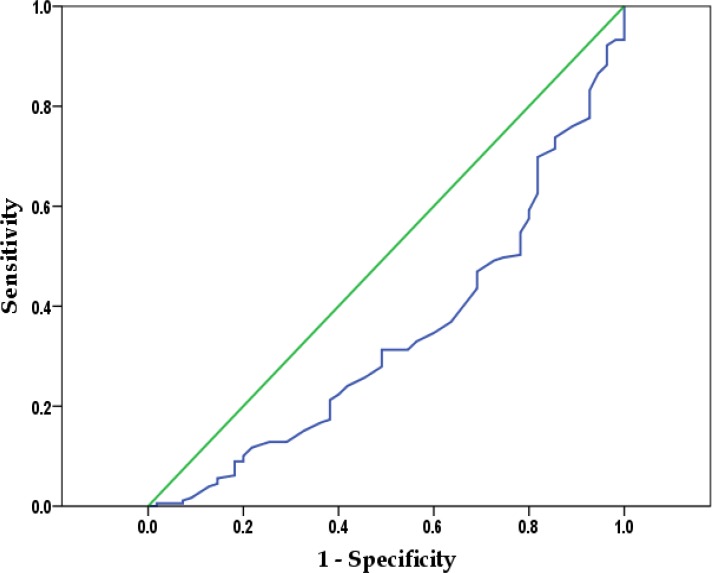
ROC curves of FEF_25-75_ measurement in the diagnoses of airway hyper-responsiveness.

In order to determine optimal discrimination threshold values for FEF_25-75_, ROC curve was used, but a cut-off point for bronchial hyper-responsive could not be determined (Figure 2).

## DISCUSSION

The present study, carried out on patients with asthma like symptoms and normal pulmonary function, highlights that a drop in baseline FEF_25-75_ is associated with a rise in the number of hyper responsive patients but does not correspond with levels of AHR. Furthermore, we can only say that a smaller rate of FEF_25-75_ denotes an AHR risk factor.

We did not find a major FEF_25-75_ cut-off value to distinguish hyper-reactive from normo-reactive subjects. This means that FEF_25-75_ can only be considered an AHR risk factor and could not differentiate hyper-reactive from normo-reactive subjects.

We speculate that along with normal FEV1, FEF_25-75_ may also be clinically worthwhile in diagnosis of asthmatic patients with undesirable asthma outcomes. Further, for the majority of asthmatic patients who have a normal FEV1, other findings of spirometry measurement are associated with poor asthma outcomes, and these have important implications for clinicians and investigators looking for a suitable asthma outcome measurement (13,14).

A study performed in this respect on children with a low FEF_25-75_ and normal FEV1 showed low FEF_25-75_ was significantly associated with asthma intensification and severity and the application of steroids (4). The finding of another study confirmed that small airway dysfunction is associated with a more severe AHR, nocturnal asthma, more exacerbations, asthma induced by exercise, poor asthma control and late-phase allergic response (15). These results suggest the possible role of FEF_25-75_ as a marker of asthma severity particularly in patients with normal FEV1 and FEV1/FVC. Also, low rates of FEF_25-75_ were negatively related to FeNO value (16) suggesting that in the initial phases of the disease, distal airways as opposed to proximal ones are subject to more severe inflammation and airflow obstruction. Therefore, FEF_25-75_ rather than FEV1 and FEV1/FVC is a better marker in this regard. In these patients, methacholine challenge test can confirm an asthma diagnosis.

Currie et al. (17) evaluated asthmatic patients with borderline methacholine challenge test. They measured the patients’ AHR and showed that the rate of FEF_25-75_ in patients with moderate-to-severe AHR was significantly lower, suggesting FEF_25-75_ as a marker of asthma severity. The results of the present study are not consistent with those of other studies in which lower rates of FEF_25-75_ have an inverse relationship with airway AHR. In fact, in this study no relationship was observed between FEF_25-75_ and AHR, and higher impairment of FEF_25-75_ did not correspond to a more severe AHR.

In the present study, similar to study of Sposato et al. (18), there was a drop in FEF_25-75_ rate among patients with normal reactivity. Therefore, small airway impairment measured by a decrease in FEF_25-75_ could be considered as a useful approach to detect impairment associated with other asthma parameters such as typical symptoms, wheezing, and atopy. Reduction of FEF_25-75_ in normo-reactive subjects or hyper-reactivity apart from asthma, may be due to air pollution, occupational exposure, smoking, early stage of Chronic Obstructive Pulmonary Disease (COPD) and other factors that are still unknown.

The study limitation was the absence of long term follow up of patients for evaluating and comparing the number of asthma exacerbations and patients’ outcome.

In conclusion, patients with asthma symptoms and “normal” FEV1, FVC and FEV1/FVC, but impaired FEF_25-75_, are recommended to perform a bronchoprovocation test. Unfortunately, a significant cut-off of FEF_25-75_ could not be found to help find the distinction between hyper-reactive and normo-reactive airway in that FEF_25-75_ can be low in normo-reactive subjects. In addition, no association was found between a greater impairment of FEF_25-75_ and a more severe AHR.
